# Decreased Kidney Graft Survival in Low Immunological Risk Patients Showing Inflammation in Normal Protocol Biopsies

**DOI:** 10.1371/journal.pone.0159717

**Published:** 2016-08-17

**Authors:** Fernanda Ortiz, Rosana Gelpi, Ilkka Helanterä, Edoardo Melilli, Eero Honkanen, Oriol Bestard, Josep M. Grinyo, Josep M. Cruzado

**Affiliations:** 1 Helsinki University Hospital, Nephrology, Helsinki, Finland; 2 Bellvitge University Hospital, Department of Nephrology, Barcelona, Spain; 3 Helsinki University Hospital, Transplantation and liver surgery, Helsinki, Finland; Johns Hopkins School of Medicine, UNITED STATES

## Abstract

**Introduction:**

The pros and cons for implementing protocol biopsies (PB) after kidney transplantation are still a matter of debate. We aimed to address the frequency of pathological findings in PB, to analyze their impact on long-term graft survival (GS) and to analyze the risk factors predicting an abnormal histology.

**Methods:**

We analyzed 946 kidney PB obtained at a median time of 6.5 (±2.9) months after transplantation. Statistics included comparison between groups, Kaplan-Meier and multinomial logistic regression analysis.

**Results and Discussion:**

PB diagnosis were: 53.4% normal; 46% IFTA; 12.3% borderline and 4.9% had subclinical acute rejection (SCAR). Inflammation had the strongest negative impact on GS. Therefore we split the cases into: “normal without inflammation”, “normal with inflammation”, “IFTA without inflammation”, “IFTA with inflammation” and “rejection” (including SCAR and borderline). 15-year GS in PB diagnosed normal with inflammation was significantly decreased in a similar fashion as in rejection cases. Among normal biopsies, inflammation increased significantly the risk of 15-y graft loss (P = 0.01). Variables that predicted an abnormal biopsy were proteinuria, previous AR and DR-mismatch.

**Conclusion:**

We conclude that inflammation in normal PB is associated with a significantly lower 15-y GS, comparable to rejection or IFTA with inflammation.

## Introduction

The pros and cons for implementing protocol biopsies (PB) after kidney transplantation are still a matter of debate. Although its use in the context of randomized controlled trials is generally approved, a concrete benefit in the routine follow-up of kidney transplant patients is yet to be fully demonstrated. Therefore, graft function is usually monitored by serum creatinine, estimated glomerular filtration rate and proteinuria, whereas a kidney biopsy is indicated to ascertain the cause of graft dysfunction. The potential benefit of recognizing unexpected pathological patterns in well-functioning kidneys early enough may have an impact on graft survival (GS), as suggested almost two decades ago by Rush. [1] The treatment of acute rejection (AR) diagnosed by protocol biopsies (i.e. subclinical acute rejection, SCAR) was translated into a better survival, [2] although these results could not be reproduced in larger studies in the era of modern immunosuppression. [3,4] Furthermore, due to improvements in immunosuppression, the incidence of clinical AR has decreased and the reported rate of SCAR is very low. For these reasons histological monitoring has been recommended only for patients at high risk for developing AR, such as cross-match positive, ABO-mismatched, highly immunized or those with delayed graft function. [5]

Long-term kidney GS has not increased remarkably in the past decade and underlying processes of both immunological and non-immunological nature could be a reason for this. [6] The role of inflammation both on indication biopsies and protocol biopsies is a well-known risk factor for kidney graft loss of immunological nature, but this negative impact has been described in association to interstitial fibrosis and tubular atrophy (IFTA) or other features compatible with AR. [7,8] However, the frequency and relevance of the subtle nonspecific inflammation in the absence of other histopathological changes is not clear. The histological monitoring of the kidney grafts opens a window for tailoring treatment, such as minimization of immunosuppression in cases of normal histology. Although this measure could diminish the impact of calcineurin inhibitor-related nephrotoxicity or the adverse effects secondary to the use of steroids, lately there has been increasing concern about the negative impact of the minimization on the development of de novo donor specific antibodies leading to chronic rejection. [9]

In the present study we aimed to address the frequency of pathological findings in a large material of protocol biopsies in two European transplant centers, to analyze their impact on long-term graft survival (GS) and to analyze the risk factors predicting an abnormal histology which might help target patients who could get a benefit from this procedure.

## Material and Methods

### Patient population

Altogether 946 cross match negative ABO compatible kidney transplant patients (KT) from Helsinki University Hospital and Bellvitge University Hospital were biopsied by protocol from January 1994 to December 2011. Candidates for PB gave written consent for this procedure and further histological analysis. They should have had a stable serum creatinine concentration and uneventful follow-up during one month previous to the biopsy. Histological monitoring of kidney allografts with protocol biopsies is part of our regular follow-up protocol and for this reason neither the Helsinki University Hospital nor the Bellvitge University Hospital ethical committees require permission for this type of research. Time for PB slightly changed over the years, aimed at 6 months post transplantation in the majority of the cases, but for a short period we performed at 3 months (only in Helsinki) and in the last years at 12 months. Only one protocol biopsy per patient was considered and follow-up biopsies were not included. Urinary tract infection was ruled out. Recipients of multiple organs were not considered. We discarded 20 cases because of insufficient follow-up data and 23 cases because of insufficient histological material, leaving a total of 903 biopsies to be included in this analysis (N = 480 from Helsinki and N = 423 from Bellvitge).

Clinical data included donor and recipient demographics, type and number of transplant, panel reactive antibodies (PRA), A-, B- and DR- mismatch, occurrence of delayed graft function (DGF), cold ischemia time (CIT), clinical rejection episodes before PB (AR), time from transplantation to protocol biopsy, serum creatinine, estimated GFR with CKD-EPI [10], immunosuppression at the time of PB, proteinuria at biopsy time and during the follow up as well. The definition of proteinuria was a positive dip-stick or measured proteinuria over 150 mg/day. Patient and death-censored GS were evaluated as of December 2012. Seventy five KT had follow-up data for over 15-y, 369 KT had follow-up data for over 10 years and 218 patients had a follow-up shorter than 5 years. The data extracted from charts and local registries was coded before analysis by one of the authors (FO), given an investigation ID-number to each case to assure anonymous manipulation of the data. This research followed recommendations in “Ethical principles of research in the humanities and social and behavioral sciences and proposals for ethical review” and the Helsinki University Hospital local ethical committee recommendations.

### Histological samples

Protocol biopsies were obtained at a median time of 6.5 (±2.9) months (75% of them from 3 to 7 months, 21% from 7,1 to 12,9 months and the rest 4% up to 24 months). Two cores of tissue were obtained under ultrasound guidance with an automated gun using either 16 or 18 Gauge needles. There were no patients´ deaths or graft losses related to this procedure. The samples were processed for routine light microscopy and stained with hematoxylin eosin, periodic acid Schiff, Masson’s trichrome and silver-methenamine. The mean number of glomeruli was 10.4. Immunofluorescence analysis was performed in 57.8% of the biopsies and included staining for IgG, IgA, IgM and C3. C4d staining was available in 43% of the biopsies, and 15/388 (3.9%) cases were positive. Histological lesions were graded by experienced nephropathologists according to the Banff diagnostic categories [11–14]. The categories included in all versions of the Banff classification are depicted in Table 1.

**Table 1:** Individual histological components included in all versions of the Banff classification.

i0—No or trivial interstitial inflammation (<10% of unscarred parenchyma)	i1–10 to 25% of parenchyma inflamed
i2–26 to 50% of parenchyma inflamed	i3 - > 50% of parenchyma inflamed
t0—No mononuclear cells in tubules	t1—Foci with 1 to 4 cells/tubular cross section (or 10 tubular cells)
t2—Foci with 5 to 10 cells/tubular cross section	t3—Foci with >10 cells/tubular cross section, or the presence of at least two areas of tubular basement membrane destruction accompanied by Í2/Í3 inflammation and t2 tubulitis elsewhere in the biopsy
v0- No arteritis	v1- Mild-to-moderate intima arteritis in at least one arterial cross section
v2—Severe intima arteritis with at least 25% luminal area lost in at least one arterial cross section	v3—Transmural arteritis and/or arterial fibrinoid change and medial smooth muscle necrosis with lymphocytic infiltrate in vessel
g0—No glomerutitis	gl—Glomerulitis in tess than 25% of glomeruli
g2—Segmental or global glomerutitis in 25 to 75% of glomeruli	g3—Glomerulitis (mostly global) in more than 75% of glomeruli
ci0—Interstitial fibrosis in up to 5% of cortical area	ci1- Mild—interstitial fibrosis in 6 to 25% of cortical area
ci2—Moderate—interstitial fibrosis in 26 to 50% of cortical area	ci3—Severe—interstitial fibrosis in >50% of cortical area
ct0—No tubular atrophy	ct1- Tubular atrophy in up to 25% of the area of cortical tubules
ct2—Tubular atrophy involving 26 to 50% of the area of cortical tubules	ct3—Tubular atrophy in >50% of the area of cortical tubules
cg0—No glomerulopathy—double contours in <10% of peripheral capillary loops in most severely affected glomerulus	cgl—Double contours affecting up to 25% of peripheral capillary loops in the most affected of nonsclerotic glomeruli
cg2—Double contours affecting 26 to 50% of peripheral capillary loops in the most affected of nonsclerotic glomeruli	cg3—Double contours affecting more than 50% of peripheral capillary loops in the most affected of nonsclerotic glomeruli
cv0—No chronic vascular changes	cv1- Vascular narrowing of up to 25% luminal area by fibrointimal thickening of arteries ± breach of internal elastic lamina or presence of foam cells or occasional mononuclear cells
cv2—Increased severity of changes described above with 26 to 50% narrowing of vascular luminal area	cv3—Severe vascular changes with >50% narrowing of vascular luminal area

Definition of normal (i≥0, t0 and either ci = 0 OR ct = 0), borderline (no intimal arteritis, t≥1 and i0 or i1), acute rejection (i≥2, t≥2 and/or v≥0). In 2007 was included interstitial fibrosis plus tubular atrophy (IFTA) as a new category (mild IFTA ct≥1 AND ci≥1 in <25% of the cortical area, moderate IFTA from 26% to 50% and severe IFTA>50%). Extracted from references10 and 11.

### Statistical analysis

Results are expressed as the mean ± standard deviation or 95% interval confidence for the mean. Comparison between groups was performed by either chi-square or Kruskal-Wallis test and ANOVA for more than two groups. GS was considered the outcome variable in the present study. Kaplan-Meier was employed to analyze variables associated with GS and log rank test for group comparison. Cox regression was used to analyze histological diagnostic categories as variables associated with GS. Multinomial logistic regression analysis was performed to compare the odds ratio for clinical variables respecting the histological diagnostic groups. All P-values were two-tailed and significance was set at a level of 0.05. Statistical analysis was performed using SPSS Software (version 19.0; Chicago, IL).

## Results

### Characteristics of the study population

Characteristics of the population are displayed in Table 2. Recipients were Caucasians, the majority were 1^st^ transplants from deceased donors with low HLA sensitization. Graft allocation was based on appropriate HLA-match, being HLA-AB-mismatches ≤2 achieved in 70.6% of the cases and HLA-DR-mismatches ≤1 in 93.1%. Immunosuppression at biopsy time was the following: 91.6% were on prednisone; 18.4% were on azathioprine (AZA); 70.0% on mofetil mycophenolate (MMF); 61.6% on cyclosporine A (CyA); 27.5% on tacrolimus (TAC) and 7.4% were on m-Tor-inhibitor. Induction therapy was used in 22.5% of the patients (11.3% with basiliximab and the rest with thymoglobulin). The follow-up period ranged from 0.4 to 18.5 years and mean follow-up was 7.1 y. Overall, 5-y GS was 90%, 10-year GS was 79% and 15-year GS was 74%. Altogether 231 grafts were lost (93 returned to dialysis and 138 died with a functioning graft). The prevalence of previous clinical AR was 23.3%. The odds ratio for 15-y death censored graft loss in patients with previous clinical AR was 1.43 (95% CI 1.19–1.72, P<0.001).

**Table 2:** Demographic data, kidney function at the time of protocol.

Recipient age, (years) mean (SD)	48.5 (12.6)
Recipient sex, male/female (%)	64/36
1^st^ transplant (%)	86.7
Deceased donor (%)	98.2
Aetiology (%)	
Diabetic nephropathy	14.8
Chronic glomerulonephritis	30.5
Polycystic kidney disease	14.0
Chronic interstitial nephritis	10.5
Other specified cause	9.4
Unknown	20.8
Time in dialysis, (months) mean (SD)	27.3 (29)
Donor age, (years) mean (SD)	44.7 (15.3)
Donor sex, male/female (%)	60 /40
Cold ischemia time, (hours) mean (SD)	19.5 (5.5)
Panel reactive antibodies <10%	89.9%
Delayed graft function, Yes/No (%)	26.4 / 73.6
AB-mismatches (%) 0/1/2/3/4	6/24/41/22/7
DR-mismatches (%) 0/1/2	33.7 / 59.4 / 6.9
Clinical acute rejection previous to PB (%)	23.3
Mean serum creatinine, (μmol/L) mean (SD)	119.6 (35)

### Analysis of protocol biopsies

**Fig 1:**
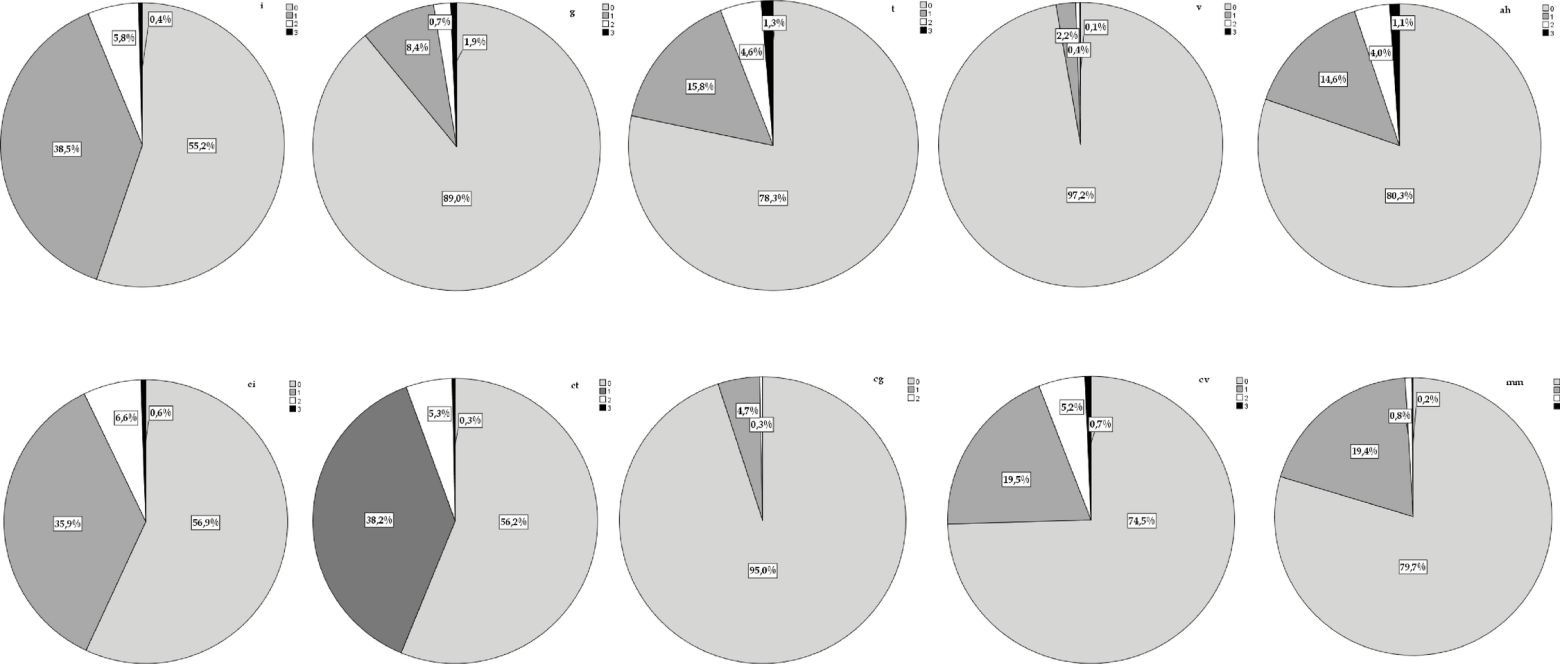
Frequency of the lesions diagnosed in PB.

The frequencies of each histological component of Banff are shown in Fig 1. Overall, 484 PB (53.6%) were normal. However, among them 361 were pristine and 123 biopsies had associated inflammatory infiltrates. IFTA was diagnosed in 257 PB (28.5%) and among them 135 had also a variable degree of inflammation. The severity of IFTA was mild in the majority of the cases (ci1 83.5%; ct1 86.4%). The prevalence of SCAR was 4.9% (N = 44). They were mainly T-cell mediated and mild (55.7% grade 1a; 28.5% grade1b; 11.4% grade 2a and concomitant grade 2b) but also subclinical AMR was diagnosed in 2 cases. Biopsies with diagnosis of SCAR had concomitantly IFTA in 37% of the cases. Borderline changes were diagnosed in 111 PB (12.3%) and among them 47 had concomitantly IFTA. Glomerulonephritis recurrence was suspected in one PB and confirmed in subsequent biopsies. This case was discarded to further analysis.

### Impact of the histological findings on graft function and GS

**Fig 2:**
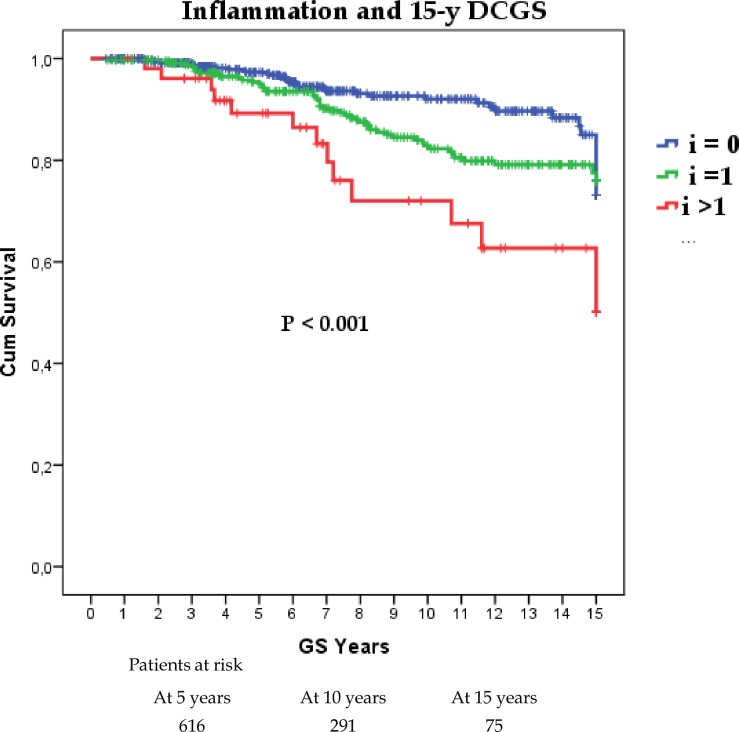
Impact of inflammation severity on death censored graft survival.

Among all the individual components of Banff, inflammation had the strongest negative impact on survival and proportional to its severity, as shown in Fig 2. The presence or the severity of interstitial fibrosis, tubular atrophy or chronic vasculopathy did not show any impact on GS (Log Rank P = 0.33; P = 0.57 and P = 0.71 respectively). The presence of glomerulitis had a negative impact on GS only when it was associated with inflammation (P = 0.04). Due to the notable impact of inflammatory infiltrates on GS we further split the normal and IFTA categories into: *a)* normal without inflammation (i0, t0, ci0, ct0) including in this category cases with *either* interstitial fibrosis (i0,t0, ci≥1, ct0) *or* tubular atrophy (i0, t0, ci0, ct≥1); *b)* normal with inflammation (i≥1, t0) and those cases including *either* interstitial fibrosis (i≥1, ci≥1, ct0) *or* tubular atrophy (i≥1, ci0, ct≥1); *c)* IFTA without inflammation (i0, t0, ci≥1 *and* ct≥1) and *d)* IFTA with inflammation (i≥1, t0, ci≥1 *and* ct≥1). Borderline and SCAR were defined according to Banff and compared to the previously mentioned categories.

The mean creatinine concentration in plasma at the time of the PB was 113 umol/L (95% CI 110–117) in PB diagnosed as normal without inflammation, while in those normal with inflammation was 119 umol/L (95% CI 112–126). In borderline cases the mean creatinine concentration was 119 umol/L (95% CI 110–127). The differences between these subgroups were not statistically different. Even for the cases diagnosed with SCAR the mean creatinine concentration was 128 umol/L (95%CI 109–149) and the P value was 0.065. The differences in graft function reached the statistical level of significance only in the cases of normal biopsies without inflammation compared with both IFTA without inflammation (P = 0.002) and IFTA with inflammation (P = 0.007), being the creatinine concentration in cases with IFTA without inflammation 127 umol/L (95%CI 121–134) and 126 umol/L (95%CI 120–131) if IFTA was associated with inflammation.

**Fig 3:**
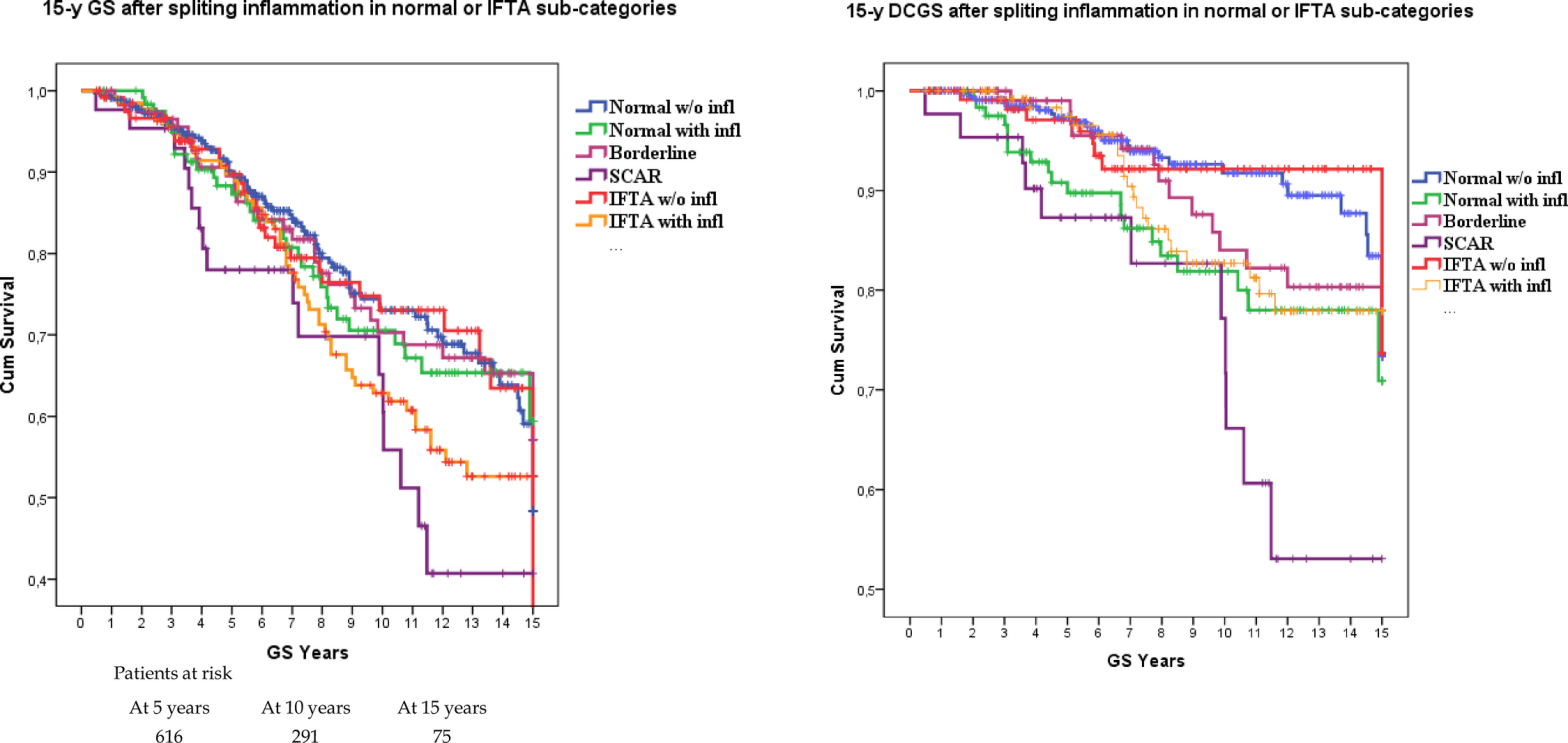
Graft survival of Banff categories split regarding the presence of inflammation. In panel A: 15-y graft survival. In panel B: 15-y death-censored graft survival.

The split of normal PB and IFTA according to the presence of inflammation showed a strong impact on both 15-y GS and 15-y DCGS, as shown in Fig 3. The best survival was observed in the groups without inflammation (both normal histology and IFTA). The worst GS was seen in cases of SCAR, but borderline category had also a weak survival comparable to normal with inflammation and IFTA with inflammation. The difference in 15-y DCGS between “normal without inflammation” and “normal with inflammation” was statistically significant (P = 0.01)

We calculated the risk for graft loss taking as variables the five groups in which the PB were divided. The reference group was “normal w/o inflammation”. In the multivariate analysis the risk for graft lost at 15 years was significantly increased in case of a normal biopsy with inflammation and rejection. The hazard ratios are depicted in Table 3.

**Table 3:** Cox regression was use for investigating the effect of histological categories diagnosis upon 15-y death censored graft survival.

Covariates	Univariate			Multivariate		
	HR	95% CI	P value	HR	95% CI	P value
**Normal WITH inflammation**	1.96	1.37–2.55	0.023	2.01	1.42–2.59	0.018
**IFTA w/o inflammation**	0.94	0.17–1.71	0.888	------		
**IFTA WITH inflammation**	1.58	0.74–2.09	0.164	------		
**Rejection**	1.819	1.27–2.37	0.030	1.78	1.22–2.33	0.038

The reference group was “normal without inflammation”, being the other categories´ hazard ratios calculated first in all categories and afterwards keeping those statistically significant from the univariate analysis.

### Factors predicting survival

For this analysis we unified borderline and SCAR into “rejection” and compared to the other groups. As shown in Table 4, PRA percentage, CIT occurrence or DGF or male sex did not differ among categories. Both donor age and time to PB were higher in IFTA, as expected. We observed a higher number of HLA AB-mismatches in IFTA without inflammation and rejection groups, which was in both cases significantly higher compared to normal with inflammation. HLA DR-mismatches occurred more frequently in all subgroups with inflammation infiltrates (P<0.001). In a similar way, previous episode of AR was more frequent in all the subgroups with inflammation. There was a considerable increase in the number of patients developing proteinuria during the follow-up, being those with SCAR and borderline the subgroups with the highest increase from 14.8% to 37.7% of the patients. Notably, the “normal with inflammation” subgroup also experienced a considerable increase of proteinuria, from 8.2 to 24.4%. The patients with pristine normal biopsies had the least proteinuria both at PB (P<0.001) and at the end of the follow-up (P = 0.001). To evaluate the impact of each of these statistically significant variables on graft survival we performed a multinomial regression analysis using the “normal without inflammation” as the reference group. The results are exposed in Table 5. Of notice, a previous AR episode and HLA DR-mismatch increased the odds of having a “normal PB with inflammation” (OR 2.67 and 2.18, respectively). The presence of proteinuria at BP increased the odds of having a PB with IFTA, SCAR or borderline diagnosis. A HLA DR-mismatch also increased the odds of having a PB with rejection (OR 1.67) or IFTA with inflammation (OR 1.79).

**Table 4:** Multinomial logistic regression analysis was performed to compare the odds ratio for demographic, immunological, clinical and biochemical variables respecting the 5 histological diagnostic groups.

Normal w/o inflammation N = 361	Normal w/ inflammation N = 123	IFTA w/o inflammation N = 122	IFTA w/ inflammation N = 297	Rejection N = 160	P
***Donor age (years)*** mean (95%CI)					
43.8 (42.2–45.4)	43.2 (40.5–45.9)	50.0 (47.3–52.6)	45.6 (43.2–47.9)	43.5 (40.0–46.1)	***P< 0*.*01*** ^***c-a*,*b*,*e***^
***Panel reactive antibodies (%)*** mean (95%CI)					
4.5 (2.9–6.2)	4.8 (1.9–7.7)	3.2 (1.4–5.1)	5.1 (2.2–7.9)	2.9 (1.2–4.6)	P = 0.62
***Cold ischemia time (hours)*** mean (95%CI)					
19.6 (19.1–20.2)	18.9 (17.9–19.9)	19.6 (18.5–20.6)	19.9 (19.2–20.7)	19.1 (18.1–19.9)	P = 0.44
***Time to biopsy (months)*** mean (95%CI)					
6.3 (6.0–6.5)	6.5 (6.0–7.0)	7.1 (6.4–7.8)	7.4 (6.9–8.0)	5.9 (5.5–6.3)	***P = 0*.*01*** ^***c-e***^ ***P< 0*.*01*** ^***d-a*,*e***^
***Plasma creatinine at biopsy (SD)***					
113.6 (32.4)	119.5 (37.7)	127.5 (37.7)	125.8 (34.6)	124.1 (28.1)	***P = 0*.*004*** ^***a-c***^ ***P = 0*.*013*** ^***a-d***^
***Donor sex (male)*** N = 580 (64.2%)					
238 (64,2)	77 (62.6)	82 (67.2)	86 (63.7)	97 (59.9)	P = 0.66
***Delayed graft function (yes)*** N = 237 (26.4%)					
85 (23.7)	38 (30.9)	36 (29.8)	46 (34.1)	32 (19.8)	P = 0.35
***HLA AB-mismatches >2*** N = 266 (29.5%)					
103 (28.5)	22 (17.9)	46 (37.7)	29 (21.5)	66 (40.7)	**P<0.001** ^**b-c.e**^
***HLA DR-mismatches >0*** N = 601 (66.6%)					
212 (58.7)	96 (78.0)	75 (61.5)	101 (74.8)	117 (72.2)	**P<0.001** ^**a—b,d,e**^ **P<0.001** ^**b,c**^
***Clinical acute rejection previous to PB (yes)*** N = 210 (23.3%)					
48 (13.3)	40 (32.5)	19 (15.6)	45 (33.3)	58 (35.8)	**P<0.001** ^**a- b,d,e**^ **P<0.001** ^**b-c**^
***Proteinuria at biopsy (yes)*** N = 87 (9.7%)					
18 (5.1)	10 (8.2)	18 (15.0)	17(12.6)	24 (14.8)	**P<0.001** ^**a- c,d,e**^
***Proteinuria during follow-up (yes)*** N = 215 (24%)					
63 (17.6)	30 (24.4)	29 (24)	32 (23.7)	61 (37.7)	**P = 0.001** ^**a-e**^

Rejection category includes borderline. Presence of proteinuria meant a positive dip-stick or measured proteinuria over 150 mg/day. a) Normal without inflammation. b) Normal with inflammation. c) IFTA without inflammation. d) IFTA withy inflammation. e) Rejection.

**Table 5:** Multinomial logistic regression analysis results for prediction the probabilities of each histological categories given a set of independent variables selected from the logistic regression analysis.

	*B*	*Sig*.	OR	*95% CI for OR*	
				Lower Bound	Upper Bound
**Normal with inflammation**					
Donor age	– 0.002	0.785	0.99	0.98	1.01
Previous AR	**0.985**	**<0.001**	**2.67**	**1.61**	**4.44**
Proteinuria at PB	0. 590	0.161	1.80	0.794	4.12
HLA AB-mismatch >2	– 0.464	0.085	0.62	0.37	1.06
HLA DR-mismatch ≥1	**0.780**	**0.002**	**2.18**	**1.33**	**3.57**
**SCAR + borderline**					
Donor age	0.000	0.95	1.00	0.99	1.02
Previous AR	**1.256**	**<0.001**	**3.51**	**2.20**	**5.59**
Proteinuria at PB	**1.033**	**0.004**	**2.72**	**1.38**	**0.53**
HLA AB-mismatch >2	**0.642**	**0.002**	**1.89**	**1.25**	**2.86**
HLA DR-mismatch ≥1	**0.515**	**0.017**	**1.67**	**1.09**	**2.25**
**IFTA w/o inflammation**					
Donor age	**0.027**	**<0.001**	**1.03**	**1.01**	**1.04**
Previous AR	0,273	0.377	1.31	0.76	2.41
Proteinuria at PB	**0,911**	**0.013**	**2.48**	**1.21**	**5.08**
HLA AB-mismatch >2	0.401	0.081	1.49	0.95	2.34
HLA DR-mismatch ≥1	0.088	0.692	1.09	0.71	1.69
**IFTA with inflammation**					
Donor age	0.008	0.253	1.01	0.99	1.02
Previous AR	**1.099**	**<0.001**	**3.00**	**1.84**	**4.89**
Proteinuria at PB	**0.954**	**0.009**	**2.59**	**1.26**	**5.32**
HLA AB-mismatch >2	-0.282	0.256	0.75	0.46	1.22
HLA DR-mismatch ≥1	**0.583**	**0.012**	**1.79**	**1.13**	**2.82**

Normal without inflammation was used as reference category. AR: acute rejection; PB: protocol biopsy. OR: odds ratio. In bold statistically significant values.

### Changes in the immunosuppression after PB

In all, 81% of the patients were on triple immunosuppressive treatment at biopsy. CyA, MMF and steroids was the most common combination (40%), followed by TAC, MMF and steroids (23%). Only 4% were on TAC, AZA and steroids and 14% on CyA, AZA and steroids. The choice of TAC over CyA was influenced by the recipient higher immunological risk (re-transplantation, high PRA, HLA mismatches) and vintage (change in the choice of CNI towards TAC in the recent era).

From 553 patients who were on CyA at PB, 34 (6.1%) were switched to TAC by the end of the 2^nd^ year. Steroids were discontinued by the end of the 2^nd^ year in 218 patients under CyA treatment (39.4%). TAC was used by 246 patients at PB and 115 of them (46.7%) were off steroids by the 2^nd^ year. Overall, after the PB steroids were discontinued in 51.1% of the patients with normal PB without inflammation, while the figures for the other groups were 48.4% in normal with inflammation, 35% for IFTA without inflammation, 23.9% for IFTA with inflammation and 27.2% for the rejection group (P<0.001). The continuation of steroids over the 2^nd^ year after did not have a statistical significant impact on the graft survival in any of the groups (P = 0.29). The modifications in the immunosuppression were not associated with the outcome in any of the four histological groups.

## Discussion

The most important finding in this study was that kidney PB with trivial inflammation between 11–25% of unscarred parenchyma diagnosed otherwise as normal had a reduced long term survival comparable to PB with rejection. Our results highlight the fact that “innocent” mild infiltrates in otherwise normal biopsies may have a deleterious effect and they should be considered relevant in clinical practice. We observed that inflammation is a frequent finding in protocol biopsies, affecting 45% of them. Furthermore, of the 484 biopsies diagnosed as normal, 25% showed inflammation. In a previous study focused also on PB obtained at 1 to 4 months after KT the frequency of inflammation was 19%. These patients had more IFTA in a control biopsy obtained after one year compared to those without inflammation. [15] Mengel et al came to a similar conclusion in their study: if the infiltrates persisted in successive biopsies graft prognosis was worse, although the diagnosis of inflammation included foci of inflammation in scarred areas. [16] The DeKAF study also suggested that the inclusion of inflammation in scarred areas in the assessment of biopsies may provide better prognostic information. [17] In our material the PB were classified using the latest version of the Banff classification contemporary with the time when the PB was taken, where inflammation was scored only in non- scarred areas. We might suggest that using the total inflammation score proposed recently by the Banff working group the frequency of truly normal biopsies could have been even lower. Even though we do not have serial biopsies to evaluate the evolution of the inflammatory infiltrates, we found evidence that the mere presence of inflammation in a well-functioning graft may provide prognostic value to the physician.

The inflammation in normal biopsies was associated even with a worse prognosis than IFTA alone. This observation is in agreement with previous reports where mild fibrosis did not affect the prognosis compared to normal biopsies, contrarily to IFTA with inflammation. [18] In the past years the Banff classification gave a great emphasis to interstitial fibrosis and tubular atrophy, as it has been linked to graft dysfunction. [19,20] Our data suggests that IFTA, although associated with elevated serum creatinine, does not necessarily worsen the kidney graft prognosis, but the most relevant histological risk factor is inflammation. Our results could be partially affected by the mild degree of interstitial fibrosis and tubular atrophy observed in our material. Both inflammation and IFTA are quite unspecific markers since they can be associated with immunological damage but also with non- immunological insults like calcineurin inhibitor toxicity and infection.

The detrimental effect of previous AR episode and persistent inflammation on protocol biopsies obtained after the 1^st^ year from transplantation has been very well described before [5,8,15,19,21–24]. In the case of proteinuria, the incidence after 1 year from transplantation varies between 11 and 45%. [25] Others suggested that proteinuria is detrimental only when associated to AR. [25–27] We observed that the prevalence of proteinuria at the time of the biopsy was 9.5% when we defined a positive value as low as over 150 mg/day or 1+ positive urine dip-stick. This low threshold for proteinuria is the reason why 5% of patients with normal biopsies had proteinuria, although the lowest. The presence of inflammation increased the frequency of proteinuria in our study cohort also in “normal with inflammation”, and IFTA. This is in dissidence with the previous reports in the literature associating it mostly to rejection. Proteinuria increased remarkably during follow-up in all groups. Again, the lowest number of patients with proteinuria belonged to the “normal w/o inflammation” group and the highest number of patients to the group “rejection”. Our results are in concordance with those reporting that the persistence or appearance of proteinuria after KT is a marker of graft damage and related to worse GS [28–30] and patient survival. [31]

One of the most argued clinical issues in PB policy is why take a biopsy from a well-functioning graft. It is well known that serum creatinine concentration is a poor indicator of graft histology. [32] We analyzed the graft function in each histological category and we found that patients diagnosed with IFTA had a statistically higher creatinine than all the other groups. These patients received grafts from older donors, raising the possibility that, at least partly, the chronic changes came along with the donor. Unfortunately, we do not have an implant biopsy from all our patients to focus on this aspect. Remarkably, normal and borderline cases had a similar creatinine concentration, while SCAR and IFTA cases showed a higher creatinine concentration. This highlights the unreliability of serum creatinine as a surrogate of histology.

Along with a decline in the incidence of previous clinical AR, SCARs have also become more infrequent. The incidence of SCAR and borderline changes is our study was 4.9% and 12.3%. Many researchers combined borderline changes with SCAR due to a comparable GS [18,33] and similar molecular phenotyping [34] of both entities. Based on these considerations we decided to join both entities for a group comparison with normal and IFTA. We analyzed which variables could predict the histological categories and we did not find CIT, DGF, PRA or male gender to be relevant. Although in previous studies biopsies from patients on CyA showing more inflammation than those on TAC [23], we decided not include CNI-choice in the multivariate analysis due to the fact that the choice of CNI was not random and it was influenced by the patient´s immunological risk and vintage. On the contrary, donor age, proteinuria at biopsy, HLA DR-mm ≥1, HLA AB-mm >2, time from transplantation and previous AR episode were the covariates that might discriminate patients at a higher risk for abnormal histology. These results could help selecting patients who could eventually benefit from a PB, avoiding this procedure in those with no risk factors.

Major limitations of our study are its retrospective design, the lack of donor specific antibody monitoring and adherence to medication. It´s worth to highlight that the role of non-adherence to medication was not studied during this study period, and data about its possible impact on the appearance of donor specific antibodies is not available. Finally follow-up biopsies were not available to ascertain the effect of late AR episodes. The high number of patients followed up for up to 18 years after transplantation gives a particular strength to the analysis.

In conclusion, this study showed that 60% of the PB were pathologic. The most relevant abnormality was the presence of inflammation, which was the main factor affecting GS. Particularly trivial inflammation affecting otherwise normal biopsies was associated to poor prognosis. A previous AR episode, the presence of even low grade proteinuria and DR mismatch were the variables that predicted the best an abnormal biopsy. A new look at the inflammation scoring in the Banff classification might contribute to a better stratification of patients at risk of graft loss.
